# USE OF MAGNETIC RESONANCE IMAGING TO DIAGNOSE BRACHIAL PLEXUS INJURIES

**DOI:** 10.1590/1413-785220182602187223

**Published:** 2018

**Authors:** BRUNO AZEVEDO VERONESI, MARCELO BORDALO RODRIGUES, MARINA TOMMASINI CARRARA DE SAMBUY, RODRIGO SOUSA MACEDO, ÁLVARO BAIK CHO, MARCELO ROSA DE REZENDE

**Affiliations:** 1. Hand and Microsurgery Group, Instituto de Ortopedia e Traumatologia, Hospital das Clinicas HCFMUSP, Faculdade de Medicina, Universidade de São Paulo, São Paulo, SP, Brazil.; 2. Radiology Department, Instituto de Ortopedia e Traumatologia, Hospital das Clinicas HCFMUSP, Faculdade de Medicina, Universidade de São Paulo, São Paulo, SP, Brazil.

**Keywords:** Brachial plexus/injuries, Brachial plexus neuropathies/diagnosis, Magnetic resonance imaging., Plexo braquial/lesões, Neuropatias do plexo braquial/ diagnóstico, Imagem por ressonância magnética.

## Abstract

**Objective:**

To compare magnetic resonance imaging and intraoperative findings in patients diagnosed with traumatic injury to the brachial plexus.

**Methods:**

Patients with a diagnosis of traumatic injury to the brachial plexus admitted to the hand and microsurgery outpatient consult of the Hospital das Clínicas at the University of São Paulo were selected during December 2016. A total of three adult patients with up to six months of injury who underwent surgical treatment were included in the study. A diffusion-weighted sequence magnetic resonance protocol and fluid-sensitive volumetric reformatting sequence were applied. The magnetic resonance results were compared with the diagnoses obtained from the injuries observed during the surgery. The study was double-blind (surgeon and radiologist).

**Results:**

A descriptive correlation was found between the magnetic resonance imaging results and the diagnostic findings from the surgeries, for both pre- and post-ganglionic injuries.

**Conclusion:**

Magnetic resonance imaging has shown to be a promising diagnostic method in preoperative assessment of brachial plexus lesions; it is less invasive than other common methods, showing not only avulsion lesions but also localized postganglionic lesions in the supra- and infraclavicular region. Level of Evidence III; Diagnostic studies - Investigating a diagnostic test.

## RESUMO

**Objetivo:**

Comparar resultados de ressonância magnética e achados intraoperatórios de pacientes com diagnóstico de lesão traumática do plexo braquial.

**Métodos:**

Foram selecionados pacientes com diagnóstico de lesão traumática do plexo braquial admitidos no ambulatório de mão e microcirurgia do Hospital das Clínicas de São Paulo no mês de dezembro de 2016. Foram incluídos pacientes adultos com até seis meses de lesão que seriam submetidos a tratamento cirúrgico, totalizando três para o estudo. Foi aplicado um protocolo de ressonância magnética com sequência ponderada em difusão e sequência com reformatação volumétrica fluido-sensível. Os resultados da ressonância magnética foram comparados com os diagnósticos das lesões obtidos na cirurgia. O estudo foi duplo-cego (cirurgião e radiologista).

**Resultados:**

Houve correlação em termos descritivos entre os laudos das ressonâncias magnéticas e os achados diagnósticos das cirurgias, tanto para as lesões pré como pós-ganglionares.

**Conclusão:**

A ressonância magnética mostrou ser um método diagnóstico promissor na avaliação pré-operatória das lesões do plexo braquial, sendo menos invasiva que os outros métodos mais utilizados, permitindo o estudo não somente das lesões por avulsão, mas também das lesões pós-ganglionares localizadas na região supra e infraclavicular. Nível de Evidência III; Estudos diagnósticos - Investigação de um exame para diagnóstico.

## INTRODUCTION

Brachial plexus injury is considered the most serious neural alteration of the limbs, and is a significant challenge for those who treat it.^1^ Traumatic injuries in adults are mostly caused by traction on the neck and shoulder in high-energy accidents.^2^ The exponential increase in cases of brachial plexus injury in Brazil, especially in large cities, is associated with car and motorcycle accidents, which correspond to 80-90% of cases.^2^ ^,^ ^3^

Several classifications have been proposed for traumatic injuries to the brachial plexus. For decision-making and prognosis, they are commonly classified into three categories according to anatomical parameters: pre-ganglionic (root avulsion), post-ganglionic (rupture or injury in the continuity of the nerve), or a combination of both.^4^ ^,^ ^5^

In this context, electrodiagnostic and imaging tests have become important tools in anatomical location of these injuries. Currently, the most commonly used imaging methods are myelography, computerized myelotomography, and magnetic resonance imaging (MRI).^5^ ^-^ ^10^ The first two have the disadvantage of being invasive examinations involving considerable risk due to radiation exposure and potential adverse reaction to the contrast material, as well as poor differentiation of the tissues adjacent to the neural injury.^11^ ^,^ ^12^

Specifically in relation to the brachial plexus, MRI should be performed according to a specific protocol to obtain slices and images that can be used to stage the injuries. One differential sequencing technique is diffusion-weighted MRI of the brachial plexus, also called diffusion-weighted neurography. This sequence permits greater contrast between the nerve structures and surrounding tissue, enhancing the image of long stretches of the brachial plexus nerves, revealing both the morphological characteristics of the structures involved (caliber, continuity, and relationship with adjacent bone and muscle structures) and the pathological characteristics of the nerves (fibrosis, inflammation, and edema). When several planes are combined, the high contrast of diffusion-weighted neurography can provide a three-dimensional evaluation, permitting better location of the lesions.^13^ ^-^ ^17^

The objective of this study is to conduct a comparative assessment between preoperative diagnosis using MRI with a specific technique for the brachial plexus and intraoperative findings.

## MATERIALS AND METHODS

The study protocol was approved in advance by the institutional review board (process 10612/13).

Patients included in the study were adults (above 18 years) with clinical diagnosis of brachial plexus injury with traumatic etiology who were admitted to the outpatient hand and microsurgery clinic at the Institute of Orthopedics and Traumatology in the Hospital das Clínicas de São Paulo during December 2016, with up to six months of injury and indication for surgical treatment. The exclusion criteria were patients requiring sedation or with contraindications to MRI.

Three patients were included, all men with an average age of 30.66 years, all victims of motorcycle accidents. All patients signed an informed consent form.

In addition to the preoperative clinical evaluation, the patients underwent MRI to obtain brachial plexus imagery using a specific diffusion-weighted sequencing protocol and sequencing with fluid-sensitive volumetric reformatting. The examinations were performed using a GE HDXT 1.5-Tesla device (GE Medical Systems, Milwaukee, Wisconsin, USA), 12.0 version software, and 33mT/min gradients.

The surgeries were performed by the same team, and the dissection stages followed a standard sequence. Dissection was performed using the supra-clavicular transverse approach (two centimeters above the clavicle), where the roots and trunks are easily accessed. After making the incision in the skin and subcutaneous tissue, the omohyoid muscle was dissected and sectioned, providing an important anatomical landmark of the topography of the proximal brachial plexus and providing access to the plane of the roots and trunks. Generally, the first element of the brachial plexus that is seen is the upper trunk. The C5 root is smaller, vertical, above and to the side of the C6 root. The C7 root is below the transverse cervical artery, more horizontal than C6. The C8 root is located just above and behind the subclavian artery, which lies directly above the T1 root. 

The intraoperative evaluation was meant to diagnose and classify the lesions as avulsion (solution of complete continuity of a pre-ganglionic root), rupture (solution of complete continuity of a post-ganglionic segment), or lesion in continuity (focal thickening, without solution of macroscopic continuity of the nerve).

Intraoperative decisions related to the treatment of brachial plexus lesions were not influenced by the study.

The study was conducted in a double-blind manner: the surgeons did not have access to the preoperative MRI imaging, and the radiologists had no access to the surgical findings.

A brachial plexus inventory was created and completed by the surgeon and the radiologist. These data were compared to establish the degree of correlation between them.

## RESULTS

A close descriptive correlation was seen between the MRI reports using the technique in question and the diagnostic findings from the surgeries of the three patients.

### Patient 1

**Figure 1:**
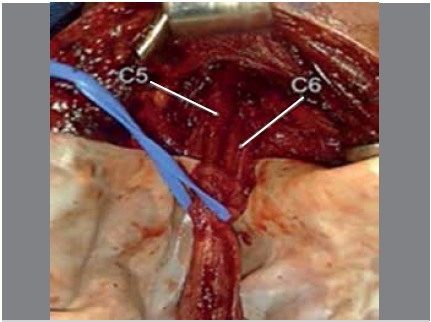
Intraoperative photo, patient 1. Identification of C5 and C6 roots forming the upper trunk, where neuroma in continuity was observed.

intraoperative findings: C5 and C6 roots and neuroma were identified in the continuity of the upper trunk. (Figure 1)

**Figure 2:**
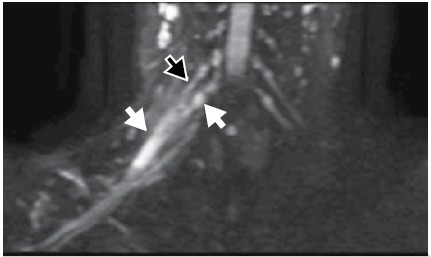
Magnetic resonance imaging, patient 1. Diffusion-weighted neurography - reconstruction in the coronal plane: Thickening and increased signal of the C5 roots (black arrow) and C6 roots (tip of arrow) and upper trunk (white arrow), corresponding to the lesion in continuity.

Description of MRI: Thickening and increased signal in the C5 and C6 roots extending to the upper trunk, compatible with post-ganglionic lesion in continuity. No signs of pre-ganglionic lesions. (Figure 2)

### Patient 2

**Figure 3:**
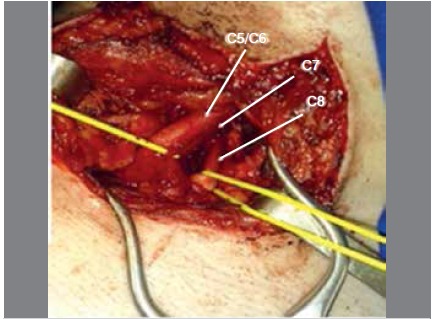
Intraoperative photo, patient 2. Visualization of C5, C6, C7, and C8 roots. Neuroma observed in the continuity of the upper trunk.

intraoperative findings: Neuroma identified in the continuity of the upper trunk. (Figure 3)

**Figure 4:**
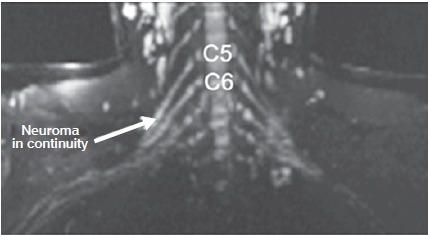
Magnetic resonance imaging, patient 2. Diffusion-weighted neurography - reconstruction in the coronal plane: Thickening and increased signal in the C5 and C6 roots extending to the upper trunk, corresponding to lesion in continuity.

Description of MRI: Thickening and increased signal in the C5 and C6 roots extending to the upper trunk, compatible with post-ganglionic lesion in continuity. (Figure 4)

### Patient 3

**Figure 5:**
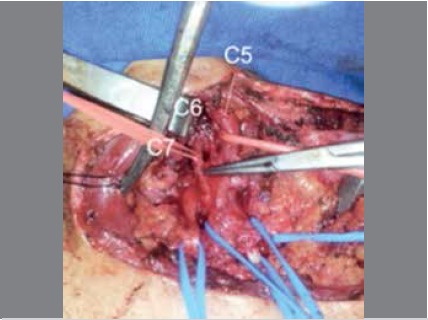
Intraoperative photo, patient 3. Neuroma in post-ganglionic region of C5 root; avulsion-type lesion of the C6 and C7 roots; neuroma in continuity at the level of the division of the lower trunk.

Intraoperative findings: Post-ganglionic neuroma-type C5 lesion in continuity; avulsion of the C6 and C7 roots; post-ganglionic lesion at the level of the division of the lower trunk, neuroma-type in continuity. (Figure 5)

**Figure 6:**
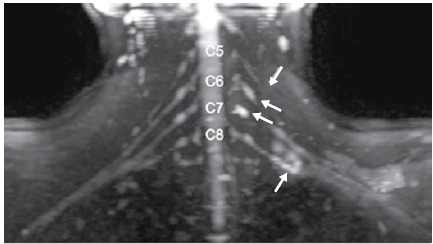
Magnetic resonance imaging, patient 3. Diffusion-weighted neurography - reconstruction in the coronal plane: Thickening and increased signal in the C5 root and region of the division of the lower trunk. Formation of pseudo-meningocele in the emergence of the C6 and C7 roots, suggestive of avulsion (pre-ganglionic lesion).

Description of MRI: Thickening and increased signal in the C5 root and region of the division of the lower trunk. Formation of pseudo-meningocele in the emergence of the C6 and C7 roots, suggestive of avulsion (pre-ganglionic lesion). (Figure 6)

## DISCUSSION

The incidence of traumatic brachial plexus injuries is increasing in Brazil, mainly due to the increase in motorcycle accidents.^3^ Consequently, it is important to identify tests which permit earlier and more accurate diagnosis to improve decision-making related to nerve reconstruction and consequently improve prognosis.

The earlier surgical reconstruction of the brachial plexus is performed (when this is indicated), the better the prognosis. In patients with symptoms suggesting favorable neuropraxis or partial lesions, watchful treatment may be an option if additional high-accuracy tests are not available to stage the lesions. In these cases, it is possible to wait for clinical improvement over time, avoiding an unnecessary surgical procedure. But in lesions with unfavorable prognoses (axonotmesis and neurotmesis), time is a decisive factor in surgical reconstruction. In these cases, earlier staging of lesions and indication for surgery provides better reconstructive planning and prognosis.^4^

Topographic knowledge of the lesions prior to surgery is important to avoid unnecessary dissections. Consequently, if the type and location of the lesions have not been precisely determined before surgery, supra- and infraclavicular approaches may be performed for this purpose, increasing morbidity and surgical time.

Currently, the most commonly used imaging exams are myelography, computerized myelotomography, and MRI using the conventional technique. The first two are more accurate in diagnosing root avulsion, but do not offer good definition of the brachial plexus as a whole. Furthermore, these tests present higher morbidity because of the use of intrathecal contrast. Conventional MRI, in turn, does not provides images with the definition needed for correct staging of the lesions.^11^

Therefore, when MRI with diffusion-weighted sequences and volumetric reformatting of fluid-sensitive sequences is applied to traumatic lesions of the brachial plexus it provides new diagnostic perspectives, with sensitivity and specificity similar to those of computerized myelotomography for diagnosing pre-ganglionic lesions (avulsion) and greater accuracy for post-ganglionic lesions.^11^ This technique provides greater contrast between the nerves and adjacent tissues, improving the image of the nerve pathways in the brachial plexus. Adding this sequence does not extend the examination excessively, only by approximately 5 minutes. When combined with the other sequences and planes of conventional MRI of the brachial plexus, this examination can provide a more accurate assessment without increasing morbidity.

In this study, we used a macroscopic classification of neural injury to compare with the MRI findings. Among the classifications, lesions in continuity (considered when neural thickening is observed during the surgery and when thickening and high signal are seen in MRI) may not have a good functional correlation and may exhibit varying degrees of axonal involvement ranging from neuropraxis to neurotmesis.

This is a preliminary study carried out over a short period of time, which explains the low number of patients evaluated. Studies with larger numbers of patients should be performed in order to confirm these data.

The study showed that the MRI technique described has good accuracy in diagnosing lesions of the brachial plexus throughout its entire extension, allied with lower morbidity.

## CONCLUSIONS

The specific MRI technique described herein is a promising diagnostic method for brachial plexus lesions, and provides good accuracy in identifying the type of lesion and topography. Unlike other imaging methods, it not only permits visualization of whether avulsion is present, but also studies the entire length of the brachial plexus. It can consequently help the medical team select treatment (conservative or surgical), make decisions about the most appropriate time to operate, and select the surgical technique, reducing operative time and thus allowing better prognosis for these patients.
